# Grading obstructive lung disease using tomographic pulmonary scintigraphy in patients with chronic obstructive pulmonary disease (COPD) and long-term smokers

**DOI:** 10.1007/s12149-014-0913-y

**Published:** 2014-10-15

**Authors:** Marika Bajc, Hanna Markstad, Linnea Jarenbäck, Ellen Tufvesson, Leif Bjermer, Jonas Jögi

**Affiliations:** 1 Department of Clinical Physiology and Nuclear Medicine, Department of Imaging and Physiology, Skåne University Hospital and Lund University, 22185 Lund, Sweden; 2 Department of Radiology, Center for Imaging and Physiology, Skåne University Hospital and Lund University, Lund, Sweden; 3 Department of Respiratory Medicine and Allergology, Skåne University Hospital and Lund University, Lund, Sweden

**Keywords:** Ventilation/Perfusion SPECT, Pulmonary scintigraphy, Chronic obstructive pulmonary disease (COPD), Imaging interpretation criteria, Technegas

## Abstract

**Abstract:**

The severity of chronic obstructive lung disease (COPD) is defined by the degree of flow limitation measured as forced expiratory volume in 1 s, which mainly reflects impairment of large and intermediate airways. However, COPD is primarily a small airways disease. Therefore, better diagnostic tools are needed. Ventilation-Perfusion (V/P) SPECT is a sensitive method to detect obstructive lung changes but criteria for staging airway obstruction are missing.

**Purpose:**

To define and validate criteria to stage COPD using V/P SPECT.

**Method:**

74 subjects (healthy non-smokers, healthy smokers or with stable COPD) were included. All were examined with V/P SPECT in a hybrid SPECT/CT system. Spirometry was performed and patients were evaluated with the clinical COPD questionnaire (CCQ). V/P SPECT was interpreted independently. Preserved lung function (%) was evaluated. The degree of airway obstruction on V/P SPECT was graded according to newly-developed grading criteria. The degree of airway obstruction was graded from normal (0) to severe (3). The airway obstructivity-grade and degree of preserved lung function were compared to GOLD, CCQ and LDCT emphysema extent.

**Results:**

Obstructivity-grade (*r* = 0.66, *P* < 0.001) and the degree of preserved lung function (*r* = −0.70, *P* < 0.001) both correlated to GOLD. Total preserved lung function decreased in relation to higher GOLD stage. There was a significant difference between healthy controls and apparently healthy long time smokers both regarding obstructivity-grade (*P* = 0.001) and preserved lung function (*P* < 0.001). Long-time smokers did not differ significantly from GOLD 1 COPD patients (*P* = 0.14 and *P* = 0.55 for obstructivity-grade and preserved lung function, respectively). However, patients in GOLD 1 differed in obstructivity-grade from non-smoking controls (*P* = 0.02).

**Conclusion:**

Functional imaging with V/P SPECT enables standardized grading of airway obstruction as well as reduced lung function, both of which correlate with GOLD stage. V/P SPECT shows that long-term smokers in most cases have signs of ventilatory impairment and airway obstruction not shown by spirometry.

## Introduction

Chronic obstructive pulmonary disease (COPD) is a major cause of morbidity and mortality. COPD is characterized by airflow limitation and abnormal inflammatory response, most often as a result of cigarette smoking [1, 2]. The changes that occur in the lungs include increased airway resistance due to airway fibrosis, inflammation and luminal plugs, and increased compliance caused by parenchymal destruction and loss of alveolar attachments. There are also vascular changes due to inflammatory remodelling with progressive vascular occlusion and loss of vasculature in areas with emphysematous destruction. Hence, COPD is a heterogeneous condition and the clinical presentation, pathophysiology, disease progression and response to therapy vary between patients [3]. Comorbidities like heart failure, lung cancer, pulmonary vascular disease, pulmonary embolism (PE) and atherosclerosis are common [4, 5].

Today, COPD is diagnosed and graded by spirometric indices like forced expiratory volume in 1 s (FEV1), forced vital capacity (FVC) and their ratio [1]. FEV1 measures the degree of airflow obstruction, predominantly in large and intermediate airways, but provides no explanation of the underlying pathophysiology [6]. It is therefore generally accepted that FEV1 by itself cannot describe the complexity of COPD and that FEV1 cannot be used in isolation for optimal assessment and management of the disease [1, 7]. No acceptable diagnostic alternative has been established.

The focus on individualized treatment in COPD is growing and there is a need for better ways to categorize patients into different phenotypes to be able to optimize therapy, follow and predict disease progression and measure response to therapy [3]. Different imaging modalities are generating interest within the field of COPD [8].

Tomographic pulmonary scintigraphy (ventilation/perfusion single photon emission computed tomography: V/P SPECT) is a nuclear medicine investigation that gives a 3-dimensional functional map of the ventilation and perfusion of the lungs and shows how these are affected by disease. Its primary use is in the diagnosis and follow-up of pulmonary embolism (PE) [9–11]. The introduction of ultra-fine aerosols has expanded the field of application for V/P SPECT and airway obstruction is no longer considered as a general problem for PE diagnosis, which was the case for previous aerosols with larger particle size [12]. Moreover, V/P SPECT has been shown to have applications in COPD and to be more sensitive than computed tomography (CT) and FEV1 in detecting early airway changes [13–15]. It has also been shown that V/P SPECT can be used to differentiate between healthy controls and COPD patients and to grade the degree of airway obstruction in COPD [13, 16]. Furthermore, it can be used to semi-quantitatively assess the degree of lung function reduction in obstructive lung disease and PE [13, 17]. The grading of airway obstruction in previous studies was, however, not standardized.

In this study, we aimed to develop a standardized way to grade COPD using V/P SPECT based on the distribution pattern of ultra-fine aerosol in the lungs. Moreover, we wanted to validate the COPD grading system and compare it to the Global initiative for obstructive lung disease (GOLD) classification in patients with various degrees of COPD, apparently healthy long-term smokers and healthy non-smokers.

## Patients and methods

This prospective study was performed with approval from the Regional Ethical Committee in Lund, Sweden. All patients gave their informed consent of participation.

55 patients with COPD (GOLD 1, 4 patients; GOLD 2, 37 patients; GOLD 3, 13 patients; GOLD 4, 1 patient) and 14 apparently healthy current or former long-term smokers were recruited at the department of Respiratory Medicine and Allergology, Skåne University Hospital, Lund, Sweden. Five patients without any history of smoking or obstructive lung disease were also included as controls. These were recruited from patients referred for suspected PE that did not show any signs of vascular obstruction. Patient characteristics are shown in Table 1. All patients were over the age of 40 years, clinically stable and, in the case of COPD patients, without any exacerbations during the past 4 weeks prior to inclusion. Drug therapy was kept unchanged. All subjects were assessed clinically and by the clinical COPD questionnaire (CCQ), to investigate their functional and mental state and the degree of symptoms [18]. Spirometry was performed to measure FEV1, FVC, and body plethysmography was used to measure TLC (total lung capacity) and RV (residual volume). FEV_1 _%, FVC %, RV % and TLC % values were calculated as a percentage of the predicted normal values [19].

**Table 1:** Patient characteristics among healthy controls, apparently healthy long-term smokers and patients with COPD

	Controls	Healthy smokers	COPD
*N*	5	14	55
Gender (m/w)	1/4	7/7	30/25
Age (years)	63 ± 5	69 ± 3	68 ± 5
Pack-years	0 ± 0	32 ± 6	42 ± 24
BMI (kg/m^2^)	24 ± 4	26 ± 4	25 ± 5

Values are mean ± SD

*BMI* body-mass index

The participants’ degree of obstructive lung disease was also categorized in accordance with GOLD [1]. All subjects except the five non-smoking controls underwent a combined V/P SPECT/low dose CT (LDCT) examination within 48 h of spirometry. The five controls were examined with V/P SPECT but not LDCT. The data from the V/P SPECT examination were then used to assess the degree of airway obstructivity (graded 0–3), in accordance with a predefined grading system developed at the department of Clinical physiology and nuclear medicine, Skåne, Lund University hospital. How much of the total lung function that was preserved (preserved ventilation and perfusion) was also evaluated semi-quantitatively and expressed in percent (%) of the estimated total lung function.

### Clinical COPD questionnaire

The CCQ was used to measure symptoms and functional state. It has been validated for studies of clinical control in COPD patients [18]. The 10-item CCQ is self-administered and patients are instructed to recall symptoms experienced during the last 7 days.

### Spirometry

A body plethysmograph (MasterScreen Body/Diffusion; Viasys Healthcare) was used to measure FEV1, FVC, TLC and RV. Spirometry was quality controlled in accordance with the American Thoracic Society guidelines [20].

### LDCT/V/P SPECT protocol

A combined SPECT/CT (Precedence, Philips, Best, Netherlands) system with a dual head gamma camera combined with a 16 slice CT (Brilliance) was used for the V/P SPECT/LDCT examinations. The procedure started with a CT overview image and continued with diagnostic low dose CT, (120 kV, 20 mAs/slice, 16 × 1.5 collimator, 0.5-s rotation time, and pitch of 0.813). The slice thickness was 5 mm and the incremental value 5 mm. We used filtered back projection to reconstruct LDCT images.

CT was not used for attenuation correction but to co-localize the morphological and functional changes visualized in either of the two modalities and were obtained during tidal breathing. CT was also used for emphysema extent quantification, which was done by one chest radiologist specialised in CT of the thorax. CT emphysema grading was visual and semi-quantitative and expressed the total amount of emphysema as a 10th-percentile of the total lung volume. The system used to score the extent of emphysema was an adaption of the four grade scale, used in previous studies, to an estimated 10th-percentile scale [21, 22]. All transversal sections above the level of the diaphragm were assessed. Each section was evaluated individually and graded to the 10th percentage area that demonstrated emphysematous changes. The scores from all the sections were then added and this number was finally divided with the total number of sections to get the percentage of the total lung volume affected by emphysema.

The protocol for V/P SPECT followed LDCT examination.

V/P SPECT imaging was performed in accordance with the recommendations of the European Association of Nuclear Medicine (EANM) [23, 24]. Briefly, a large-field-of-view dual-head gamma-camera with a low energy, all-purpose collimator was used. Technegas (Cyclomedica Ltd, Lucas Heights, Australia) was inhaled until 30 MBq had reached the lungs, and ventilation tomography followed. After that, with the patient in a carefully maintained supine position, 120 MBq 99mTc-MAA was slowly injected intravenously. Then, perfusion tomography was performed. Acquisition was performed in a 64 × 64 matrix, zoomed to a pixel size of 6.8 mm with 128 projections over 360 degrees. Sixty-four steps, each of 10 s duration, were used for the ventilation study, and 64 steps of 5 s duration were used for the perfusion study. Reconstruction was performed using ordered subsets expectation maximization with 8 subsets and 2 iterations. All examinations were performed in a department fulfilling the requirements of ISO/IEC 17025.

### Evaluation of V/P SPECT images

V/P SPECT was evaluated by two experienced nuclear medicine physicians, blinded to other information, and interpreted according to the guidelines of the EANM [10, 23].

Ventilation images were reviewed first to evaluate two qualitative parameters:

(1) Unevenness of Technegas distribution and (2) distribution of hot-spots (i.e., deposition of inhaled radiopharmaceutical in major and intermediate conductive airways or/and peripheral hot-spots (i.e., focal deposition of Technegas in distal airways) [12, 13]. Thereafter, ventilation and perfusion images were assessed together.

Regions with reduced/absent ventilation and/or perfusion were estimated and quantified [17].

### Grading airway obstruction and lung function with V/P SPECT

**Fig. 1:**
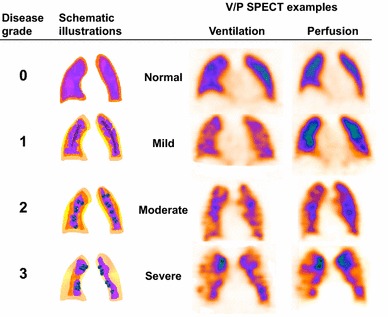
Schematic presentation of the obstructive lung disease grading system and correlating representative V/P SPECT images that shows different degrees of airway obstruction on frontal slices. *0* *Normal*, even distribution of Technegas with good peripheral penetration and without accumulation in large or small airways. *1* *Mild airway obstruction*, slightly uneven distribution with some deposition of aerosol in small and intermediate airways. Only minor areas with reduced peripheral penetration are observed. *2 Moderate airway obstruction,* deposition of Technegas in intermediate and large airways, diminished peripheral penetration with maximum accumulation in central half of the lung. *3* *Severe airway obstruction,* central deposition in large airways with severely impaired penetration of Technegas and major areas with reduced or abolished function

Qualitatively, the degree of obstructive lung disease was visually graded according to a predefined 4-point scale based on the ventilatory impairment shown by the distribution pattern of Technegas in the airways. The scale was as follows: Fig. 1.

0. Normal: even distribution of Technegas with good peripheral penetration and without accumulation in large or small airways.
1. Mild airway obstruction: slightly uneven distribution with some deposition of aerosol in small and intermediate airways. Only minor areas with reduced peripheral penetration are observed.
2. Moderate airway obstruction: deposition of Technegas in intermediate and large airways, diminished peripheral penetration with maximum accumulation in the central half of the lung.
3. Severe airway obstruction: central deposition in large airways with severely impaired penetration of Technegas and major areas with reduced or abolished function.

In addition, total preserved lung function was quantified semi-quantitatively and described in % of the total estimated lung volume. To be regarded as an area with fully preserved lung function both ventilation and perfusion had to be normal and matched.

Ventilation/perfusion defects were quantified by counting segments or sub-segments showing complete or reduced ventilation and/or perfusion defects and were expressed in % of the total lung parenchyma [13, 17, 25].

A segmental reduction or a sub-segmental total deficiency of function was attributed 1-point, and segmental total deficiency 2 points. Each lung comprises 9 segments, representing 18 points. Defects were expressed as points, which after division by 36 give the percentage of the lung that has impaired function. Thus, a theoretical total loss of function would yield 36 points.

The total sum of the V/P defects was used to estimate the extent of total reduction in lung function [17]. The physicians graded the degree of obstructive disease, if present, as mild (approximately affecting <20 % of the lung function), moderate (20–50 % approx.) or severe (>50 %). The extent of matched (reduction in *V* = reduction in *P*), mismatched (*P* < *V*) and reverse mismatched (*V* < *P*) defects were expressed as a percentage of the total lung volume. The sum of these was used to estimate the extent of total reduction in lung function. V/P SPECT images were finally reviewed according to clinical routine assessing the presence of PE, CHF or other cardiopulmonary disease [11, 12, 24, 26]. The final interpretation of V/P SPECT was based on a consensus reading.

### Statistics

The non-parametric Spearman rank correlation test was used to calculate correlations between *V*/*P* SPECT, CCQ, spirometry and emphysema extent on LDCT. The two-tailed Mann–Whitney *U* test was used for comparison of differences between groups. Statistical analysis was performed using GraphPad Prism 6.0c (GraphPad Software, CA, USA). The null hypothesis was rejected when *P* < 0.05.

## Results

Patient characteristics are shown in Table 1. All patients successfully underwent all study examinations. Table 2 shows the V/P SPECT obstructivity-grade and preserved lung function data, the emphysema extent on LDCT, spirometry values and functional state evaluation through CCQ. Table 3 gives the Spearman’s correlations between them.

**Table 2:** V/P SPECT, LDCT, CCQ and Spirometry data among healthy controls, apparently healthy long-term smokers and patients with various degrees of COPD

	Controls	Healthy smokers	COPD (GOLD grade)			
			1	2	3	4
N	5	14	4	37	13	1
V/P SPECT						
Obstructivity grade	0.0 (0.0–0.25)	1.25 (1.0–2.0)	1.75 (0.75–2.0)	2.0 (2.0–2.5)	3.0 (2.75–3.0)	3.0
Preserved lung function (%)	100 (97.5–100)	80 (69–85)	65 (47.5–94)	50 (40–65)	30 (25–40)	15
CT						
Emphysema extent (%)	N/A	0 (0–5)	2.5 (0–31)	5 (0–10)	20 (5–40)	10
CCQ	0 (0–1)	2.5 (1.5–5.5)	10 (2–20)	15 (12–22)	21 (13–26)	31
Spirometry						
FEV_1 _%	99 (94–118)	102 (92–106)	88 (64–93)	62 (55–72)	41 (34–48)	28
FVC %	110 (100–114)	96 (85–111)	107 (79–125)	99 (80–105)	81 (75–108)	74
RV %	129 (110–134)	120 (105–136)	125 (115–135)	152 (138–170)	202 (162–229)	276
TLC %	111 (104–120)	108 (98–116)	111 (99–116)	114 (101–121)	118 (112–128)	155

Values are median (interquartile range)

**Table 3:** V/P SPECT, CT, Spirometry and CCQ correlations according to Spearman

*N* = 74	Obstructivity grade	Preserved lung function (%)	Emphysema extent CT (%)	CCQ
Obstructivity grade		−0.94***	0.56***	0.58***
Preserved lung function (%)	−0.94***		−0.59***	−0.61***
Emphysema extent CT (%)	0.56***	−0.59***		0.32**
GOLD	0.66***	−0.70***	0.44***	0.63***
CCQ	0.58***	−0.61***	0.32**	
FEV1 % pred	−0.64***	0.63***	−0.42***	−0.68***
FVC % pred	−0.16	0.14	0.09	−0.32**
FEV1/FVC	−0.63***	0.64***	−0.60***	−0.60***

Values are Spearman’s correlation coefficients (*r*)

* *P* < 0.05, ** *P* < 0.01, *** *P* < 0.001

**Fig. 2:**
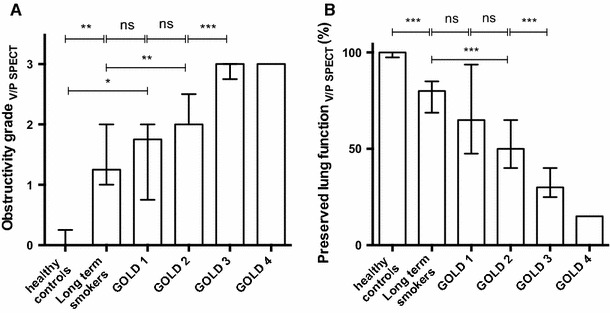
V/P SPECT Lung obstructivity grade (**a**) and preserved lung function (**b**) among healthy controls, apparently healthy long-time smokers and patients with various degrees of COPD (GOLD 1–4). *ns* no significance, *P < 0.05, **P < 0.01, ***P < 0.001. *Bars* represent median values and whiskers interquartile range

**Fig. 3:**
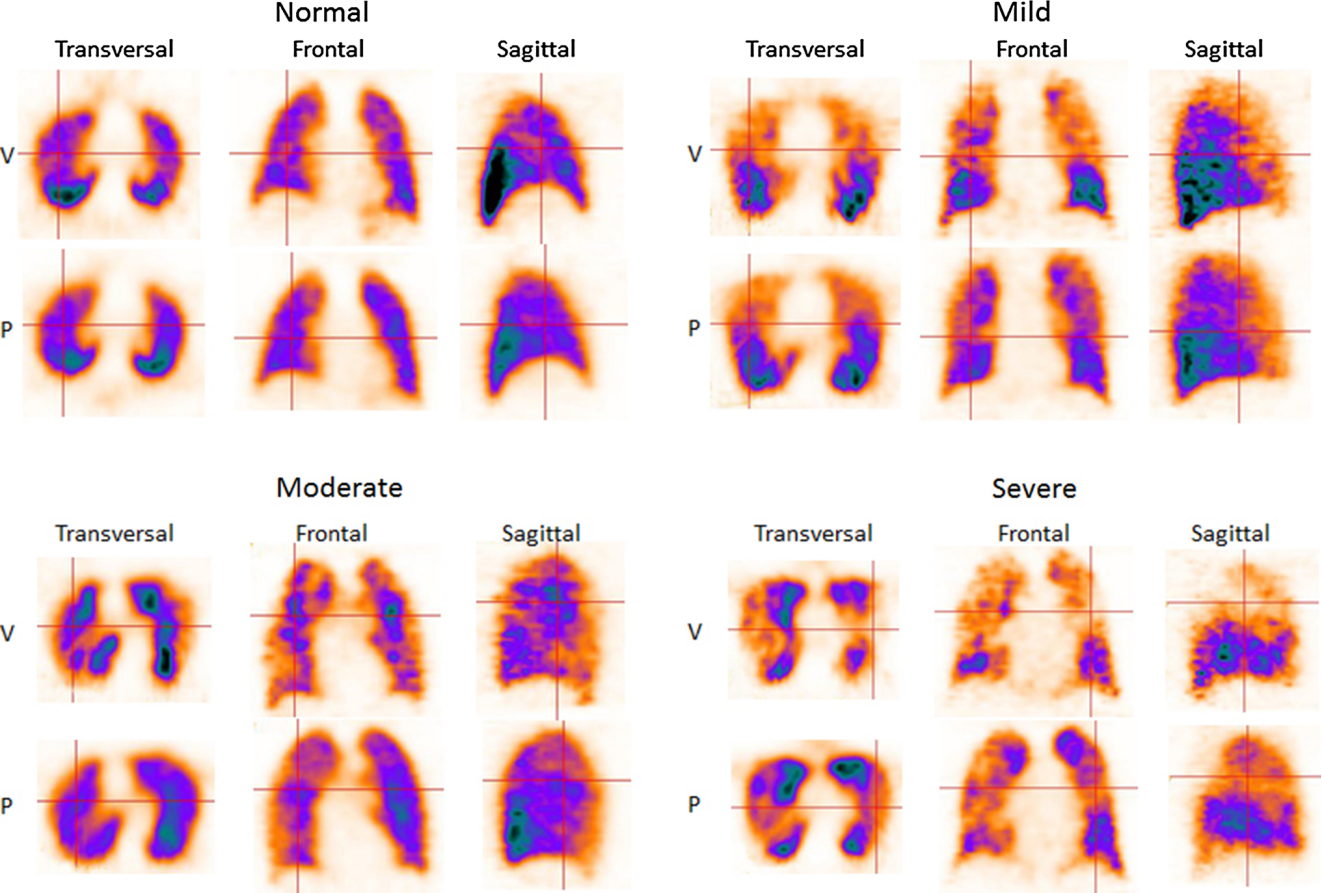
VP SPECT images showing different degrees of airway obstruction in transversal, frontal and sagittal projections

There was a moderate to strong correlation between the obstructivity-grade (*r* = 0.66, *P* < 0.001) and the degree of preserved lung function (*r* = −0.70, *P* < 0.001) compared to GOLD grade. Obstructivity-grade and the degree of preserved lung function for all groups are shown in Fig. 2. In Fig. 3, V/P SPECT images demonstrate a different degree of obstructivity in transversal, sagittal and frontal projections.

There was a significant difference between healthy controls and apparently healthy long-term smokers regarding V/P SPECT obstructivity-grade (*P* = 0.001) and preserved lung function (*P* < 0.001). Long-term smokers did not, however, differ significantly from COPD patients in GOLD 1 (*P* = 0.14 and *P* = 0.55 for obstructivity-grade and preserved lung function, respectively). Patients in GOLD 1 differed significantly in obstructivity-grade from healthy non-smoking controls (*P* = 0.02), though.

While 4 out of 5 (80 %) of the healthy controls were regarded as normal concerning airway obstructivity grade, only 1 out of the 14 (7 %) apparently healthy long-term smokers was regarded as completely normal (graded as 0). Among the COPD patients in GOLD 1 and 2 there were three individuals that had nearly normal ventilation according to V/P SPECT (obstructive-grade <1; 1 patient in GOLD 1 and 2 patients in GOLD 2). These 3 patients also showed an almost normal preserved lung function with regard to ventilation and perfusion.

Regarding emphysema extent measured on LDCT, there was only weak correlation to GOLD (*r* = 0.44; *P* < 0.001). The only significant difference observed between patient groups regarding emphysema was between patients in GOLD 3 compared to GOLD 2 (*P* = 0.05). Apparently healthy long-time smokers and GOLD 1, GOLD 1 and GOLD 2 COPD patients did not show significant differences in emphysema extent (*P* = 0.35 and 0.68, respectively).

Moderate correlations were seen between functional state measured by CCQ, and obstructivity-grade, preserved lung function, GOLD and FEV1, respectively (Table 3).

In 14 of the 55 patients with COPD (25 %), a perfusion pattern consistent with left heart failure (LHF) and pulmonary congestion was found in V/P SPECT images [26]. All patients with signs of LHF were in GOLD 2 or higher. V/P SPECT also identified vascular changes (V/P mismatch) in 16 cases (23 %) among the 69 subjects who were either long-time smokers or had COPD.

## Discussion

This study shows that V/P SPECT grading of obstructive lung disease can be standardized based on ventilation patterns in apparently healthy smokers and known COPD patients. Based on the penetration of Technegas from the center to the periphery of the lungs, four typical patterns were used to grade the degree of obstructive airway disease by two reviewers (Figs. 1, 3). A moderate to strong correlation was found when the V/P SPECT obstructivity-grade was compared to GOLD classification (Fig. 2). Interestingly, apparently healthy smokers also showed ventilatory impairment and obstructive patterns on V/P SPECT not significantly different from those seen in GOLD 1, COPD patients. Although a normal FEV1 value on spirometry testing excludes COPD, we still observed various degrees of airway obstruction among the apparently healthy long-term smokers. It is known that spirometry measures do not always correlate to the real degree of airway obstruction and clinical symptoms [27, 28]. The pathology of COPD manifests in the small airways that do not normally contribute to airflow resistance measured by FEV1. Therefore, spirometry detects changes only after airway obstruction has developed substantially. Similar patterns on lung scintigraphy regarding the penetration of Technegas and its relation to different grades of airway obstructivity were shown by Pellegrino et al. [27]. The authors suggested that the hot-spots mainly represented exaggerated deposition of Technegas occurring in the airways when narrowing is sufficiently severe to cause air flow limitations during tidal expiration.

Although three of the non-smoking controls had a completely normal ventilation/perfusion pattern on SPECT it is interesting to note that all but one of the apparently healthy long-term smokers (13/14) showed a typical obstructive pattern on V/P SPECT. This supports the opinion that spirometry is not sensitive enough to diagnose small airway disease in COPD. Among GOLD 1 and 2 COPD patients only three patients showed an almost normal ventilation pattern. This highlights the potential of V/P SPECT to detect early functional airway abnormalities that precede the decline in FEV1.

The studies performed by Pellegrino et al. [27] showed that expiratory flow limitations occurred across the lungs well before expiratory flow reserve was abolished and that methods based on mouth flow measurement are not sensitive enough to detect expiratory flow limitations. The results of the present study are consistent with the perception that airflow limitations have different, distinct aetiologies. Moreover, the extent of emphysema as measured by LDCT shows only weak correlation to GOLD grade. The severity of airflow obstruction was not predicted by the extent of emphysema. These results are in agreement with Timmins et al. [29] who predominantly analysed patients with GOLD 2 and 3.

In a study by Jobse et al. [15], where FEV1 did not correlate to the degree of emphysema, small airways disease was predicted by the ventilation/perfusion pattern on V/P SPECT. The results of the present study add to previous works by showing a method to characterize the physiological phenotypes in COPD and healthy smokers that might be clinically useful.

The preserved total lung function as quantified, by V/P SPECT, also showed a strong correlation with the level of obstructivity according to spirometry and GOLD (Fig. 2).

Comorbidities are frequent among patients with COPD [4, 30, 31] and we have confirmed the high incidence (25 %) of patients with a pattern typical for LHF and pulmonary congestion. This is also in agreement with our earlier findings [26]. Interestingly, all patients with signs of LHF were in GOLD 2 or higher. In addition, altered ventilation patterns add information regarding the functional effects of inflammatory exudate, small airway remodelling and mucus production.

The vascular response is often impaired in COPD patients and this leads to a reduced ability to adapt to ventilatory impairment by means of hypoxic vasoconstriction. In some COPD patients coagulability is increased leading to perfusion defects. Vascular defects (V/P mismatch) were a second important finding in this study and were seen in 16 cases (23 %) among the 69 subjects who were either long-term smokers or had COPD. In our earlier studies we have had the similar results [13]. The importance of the vascular component has also been stressed previously by other authors [32].

The use of V/P SPECT functional imaging in COPD patients might serve as a new tool to clarify patient’s symptoms and the heterogeneity of the disease. It may also help diagnosing comorbidities that often coexist that may have a significant impact on the prognosis. Today, according to GOLD, spirometry is used to classify patients with COPD and together with symptoms, to determine the severity of the disease. The goals are to estimate its impact on patients’ health status and the risk of future events.

Moreover, V/P SPECT allows the possibility of performing the examination in the group of patients who are experiencing chest pain or have frequent cough, which could limit the possibility to perform spirometry successfully. Furthermore, the estimate of total lung function might be of help in the choice of therapy as well as a possible way to follow-up and estimate therapy results. This needs to be further studied. Co-registration with CT images allows superior insight into the anatomic localization of lung dysfunction. However, as shown in animal studies, CT is not sensitive enough to identify early changes in COPD that can readily be visualized on V/P SPECT [33]. As smoking cessation is the best treatment of COPD, early detection is essential.

### Strengths and limitations

One of the demonstrated strengths of this study was the ability to categorize different degrees of obstructive lung disease based on the ventilation/perfusion pattern in apparently healthy smokers and known COPD patients. The second strength is the possibility to visualize co-morbidities frequently present in healthy smokers and COPD patients and the possibility to describe total lung function through estimation of ventilation and perfusion impairment. Moreover, V/P SPECT is performed during quiet breathing as opposed to spirometry. This may be a methodological advantage.

There are limitations that need to be acknowledged regarding the method of analysis. The estimation is semi-quantitative and pattern recognition subjective. LDCT reconstruction was filtered back-projection and no automatic quantification was done. Further work must be focused on development of an automated program that will facilitate accurate objective quantification.

## Conclusion

The present study shows that V/P SPECT can be used for standardized grading of airway obstruction and lung function, which both correlate to GOLD classification.

Furthermore, this study shows that even in apparently healthy long-term smokers, the majority have signs of ventilatory impairment and airway obstruction on V/P SPECT, not shown by spirometry or LDCT. This highlights the potential of V/P SPECT with its high sensitivity to detect early airway abnormalities.
